# Case Report: Hemodynamic consequences of severe supraventricular arrhythmia assessed by real-time magnetic resonance imaging in combination with electrocardiography

**DOI:** 10.3389/fcvm.2025.1596440

**Published:** 2025-06-23

**Authors:** Lena Maria Röwer, Miriam Nathalie Tappel, Mohamed Ali Goundi, Anja Müller-Lutz, Mohamed Abd El Rahman, Jana Rieke, Gerrit Steinhagen, Mathias Emmel, Dirk Klee, Frank Pillekamp

**Affiliations:** ^1^ Department of Diagnostic and Interventional Radiology, Medical Faculty and University Hospital Düsseldorf, Heinrich-Heine-University Düsseldorf, Düsseldorf, Germany; ^2^ Department of General Pediatrics, Neonatology and Pediatric Cardiology, Medical Faculty, University Hospital Düsseldorf, Heinrich-Heine-University Düsseldorf, Düsseldorf, Germany; ^3^ Independent Practitioner, Hilden, Germany; ^4^ Department of Pediatric Cardiology, Heart Center, University Hospital of Cologne, Cologne, Germany

**Keywords:** cardiac arrhythmias, atrial premature complexes, cardiovascular magnetic resonance imaging, real-time MRI, pediatrics

## Abstract

A 13-year-old asymptomatic boy presented with new-onset extrasystoles. His initial electrocardiogram (ECG) showed an irregular heart rhythm with some sinus beats but also numerous premature atrial contractions with aberrant ventricular conduction. While the initial cardiovascular magnetic resonance (CMR) study could be performed conventionally, more irregular extrasystoles impeded the follow-up study. Therefore, cardiac real-time MRI (RT-MRI) was performed in combination with a simultaneously acquired ECG, which enabled high image quality and the analysis of sinus and arrhythmic beats separately. RT-MRI volumetry of the sinus beat showed a stable systolic function in the left ventricle (LV) [LV ejection fraction of 54.3% and LV stroke volume of 61.1 ml/m^2^ (90th percentile)] and increased but stable LV volumes [LV end-diastolic volume of 112.6 ml/m^2^ (>97th percentile) and LV end-systolic volume of 51.1 ml/m^2^ (>97th percentile)]. In contrast, right ventricular (RV) function was reduced in sinus beats. In the premature contractions, RV and LV end-diastolic volume, stroke volume, and ejection fraction were lower, while the end-systolic volume was higher. In this patient with severe cardiac arrhythmias, conventional CMR could not provide adequate image quality. RT-MRI offered high image quality during free breathing and, in combination with an ECG, it provided a unique opportunity to analyze the hemodynamics of the premature beats and sinus beats as separate entities.

## Introduction

Cardiovascular magnetic resonance imaging (CMR) is the gold standard for the assessment of cardiac function and dimensions (1, 2). Conventional CMR is based on electrocardiogram (ECG)-synchronized balanced steady-state free precession (SSFP) imaging over multiple breath-holds and multiple heartbeats to acquire all necessary k-space data (3, 4). This provides high image quality during regular heartbeats. Severe arrhythmias pose a significant challenge for conventional CMR, resulting in reconstruction artifacts caused by inconsistent ECG data from multiple arrhythmic heartbeats (5). The majority of the currently used algorithms to reduce arrhythmia artifacts are based on rapid changes in the RR interval and aim to remove premature beats. Switching from retrospective to prospective gating is one of these approaches (6). This concept can improve image quality but provides information solely on the normal contractions and may miss the true diastole (5). In addition, these technical approaches are also limited in severe arrhythmias. In our case, the utilization of conventional arrhythmia suppression algorithms resulted in unfeasible breath-holding times. The change from retrospective to prospective triggering did not improve the image quality, as sinus beats and supraventricular extrasystoles could not be differentiated properly based on RR intervals. Self-gated cine magnetic resonance imaging (MRI), which extracts cardiac and respiratory motion directly from MRI data without an external ECG (7), could have been an alternative method, but given the severity of the irregularities, we would expect similar difficulties with any technique without a parallel ECG.

Recent advances in real-time MRI (RT-MRI) allow for the continuous acquisition of heartbeats with high temporal resolution during free breathing using non-linear inverse reconstruction (8, 9). This offers further unique opportunities for patients with severe arrhythmias (10).

In this case report, we show that the combination of RT-MRI with a simultaneously acquired ECG allows for cardiac imaging with high image quality and offers the unique possibility of an isolated analysis of sinus beats and arrhythmic beats, reflecting the true hemodynamic consequences of arrhythmias. This may help improve counseling for these patients.

## Case presentation

A 13-year-old asymptomatic boy who had undergone an elective follow-up study with a pediatric cardiologist (GS) because of a patent foramen ovale presented with the first occurrence of numerous premature atrial contractions with aberrant ventricular conduction. There was no history of a preceding viral respiratory infection or gastroenteritis. A physical examination was normal, with a body weight of 44 kg and height of 163 cm. The patient's blood pressure was 99/54 mmHg.

*Laboratory findings* at presentation were normal: high-sensitivity troponin T < 3 ng/L (normal <14 ng/L), creatine kinase International Federation of Clinical Chemistry and Laboratory Medicine (CK IFCC) 118 U/L (normal <269 U/L), C-reactive protein (CRP) < 0.1 mg/dL (normal <0.5 mg/dL), and NT-proBNP 60 pg/mL (normal 10–157 pg/mL).

**Figure 1:**
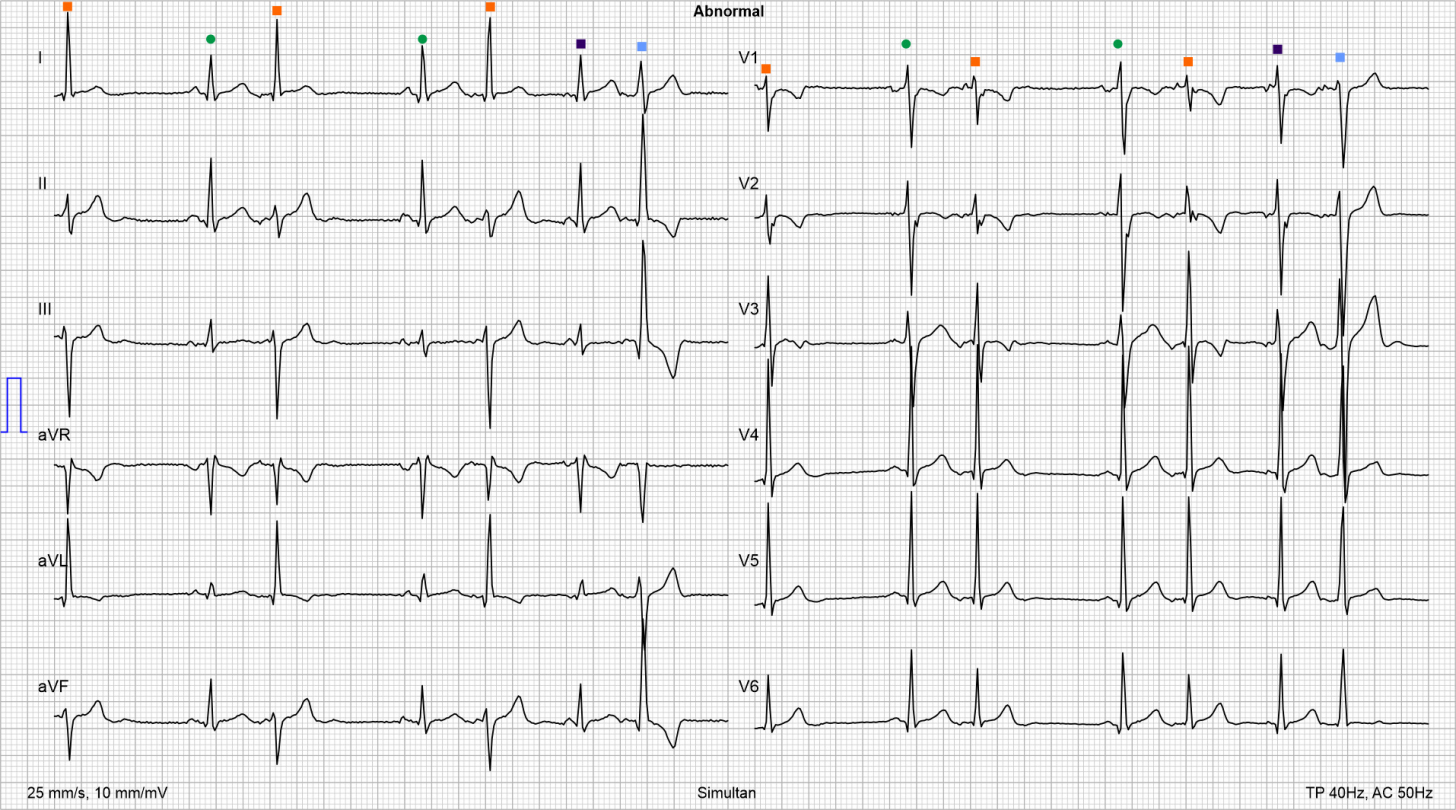
Initial ECG. Irregular heart rhythm with some sinus beats (green dots) along with numerous premature beats with different QRS complex morphologies typically preceded by a low-amplitude P wave, indicating aberrant conduction: narrow QRS complexes with a QRS vector similar to a sinus rhythm (dark purple squares) (QRS vector: ∼50°), narrow QRS complexes with a QRS vector of −20° (orange squares), and wide QRS complexes with a QRS vector of ∼−40° (blue squares).

The ECG revealed a median heart rate of 92 beats/min, consisting of a normal sinus rhythm (P ∼ 30°, QRS ∼ 50°, T ∼ 40°), a PQ of 160 ms, a QRS of 102 ms, a QT of 352 ms, and a frequency corrected QT (Bazett) of 436 ms, with multiple supraventricular extrasystoles with slightly different P wave morphologies and various QRS complex morphologies probably resulting from aberrant conduction (Figure 1).

Two-dimensional echocardiography revealed a persistent foramen ovale, mild mitral regurgitation, and a mild left ventricular (LV) dilatation [end-diastolic diameter of 52–58 mm (z-score 1.7–2.8 (11)]. The diastolic and systolic parameters were normal [left ventricular fractional shortening 37%, mitral annular plane systolic excursion (MAPSE) 19 mm, tricuspid annular plane systolic excursion (TAPSE) 29 mm].

A Holter ECG was started at the day of presentation, showing numerous extrasystoles with different QRS morphologies (7,678, i.e., 6% of all beats with broad QRS complex' according to premature ventricular contractions conducted with aberrancy; 7,204, i.e., 6% of all beats with narrow QRS complex', some couplets, 427 bigeminy with a maximal duration of 15 s, 50 trigeminy with a maximal duration of 34 s, 1,965 couplets, 91 triplets), exclusively during slow heart rates. The mean heart rate {103 bpm [>95th percentile (12)]} and the minimum heart rate {65 bpm [>95th percentile (12)]} were accelerated.

A first cardiac MRI study with the application of a contrast agent, acquired 3 weeks after the initial presentation, revealed a slightly enlarged left ventricular end-diastolic volume {106 ml/m^2^ [97th percentile (13)]} along with a borderline left ventricular ejection fraction (56%). There was no focal edema in the short tau inversion recovery (STIR) sequences. The T1- and T2-relaxation times were above the scanner- and age-specific cutoff values for myocarditis. In addition, a patchy late gadolinium enhancement was described within the left ventricular myocardium, and it was concluded that the MRI “could indicate” myocarditis. Retrospectively, these changes were at best very weak and could well have been artifacts caused by the arrhythmia.

The analysis of the first follow-up MRI, 9 months after the initial presentation, was even more severely hindered by artifacts resulting from the severely irregular heartbeats, which could not be adequately suppressed by standard arrhythmia-correction algorithms without requiring unreasonably long breath-holds. However, the results were largely consistent with those of the initial cardiac MRI study, leading to the recommendation of a beta-blocker (bisoprolol 2 × 5 mg, i.e., 0.09 mg/kg/day).

A treadmill exercise test was performed 10 months after the initial presentation, starting with a resting heart rate of 120 bpm. The boy reached a maximum heart rate of 186 bpm, showed normal oxygen consumption, and did not exhibit a single extrasystole during exertion.

The follow-up Holter ECG after the initiation of the beta-blocker showed a normalization of the mean heart rate, but a significant increase in the number of extrasystoles (31% of all beats), triggering another MRI study.

This MRI study, 14 months after the initial presentation, revealed an even greater disturbance due to the irregularity of the heartbeats, so an additional examination with RT-MRI was initiated 2 weeks later.

Cardiac RT-MRI measurements were performed on a 1.5 T clinical MRI scanner (MAGNETOM Avanto Fit, Siemens Healthineers, Erlangen, Germany; software version Syngo MR E11).

The MRI protocol started with a conventional transverse half-Fourier single-shot turbo spin-echo (HASTE) sequence to plan conventional cardiac localizers [two- and four-chamber views, left and right ventricular (RV) outflow tracts]. Thereafter, an RT-MRI sequence with balanced SSFP contrast (slice thickness, 8 mm; field of view, 320 mm × 320 mm; repetition time, 3.7 ms; echo time, 1.85 ms; flip angle, 60°; 30 frames per second) acquired a short-axis stack with 16 slices and 900 images per slice during free breathing. This was followed by a 30 s continuous real-time flow measurement at the level of the sinotubular junction of the ascending aorta and in the pulmonary trunk using a real-time phase contrast sequence (slice thickness of 6 mm; field of view, 320 mm × 320 mm; repetition time, 3.33 ms; echo time, 2.14 ms; flip angle, 12°; 30 frames per second; velocity encoding, 200 cm/s). ECG and respiratory bellows data were collected during RT-MRI acquisition using a Siemens signal recording tool, version number VE11a.

**Figure 2:**
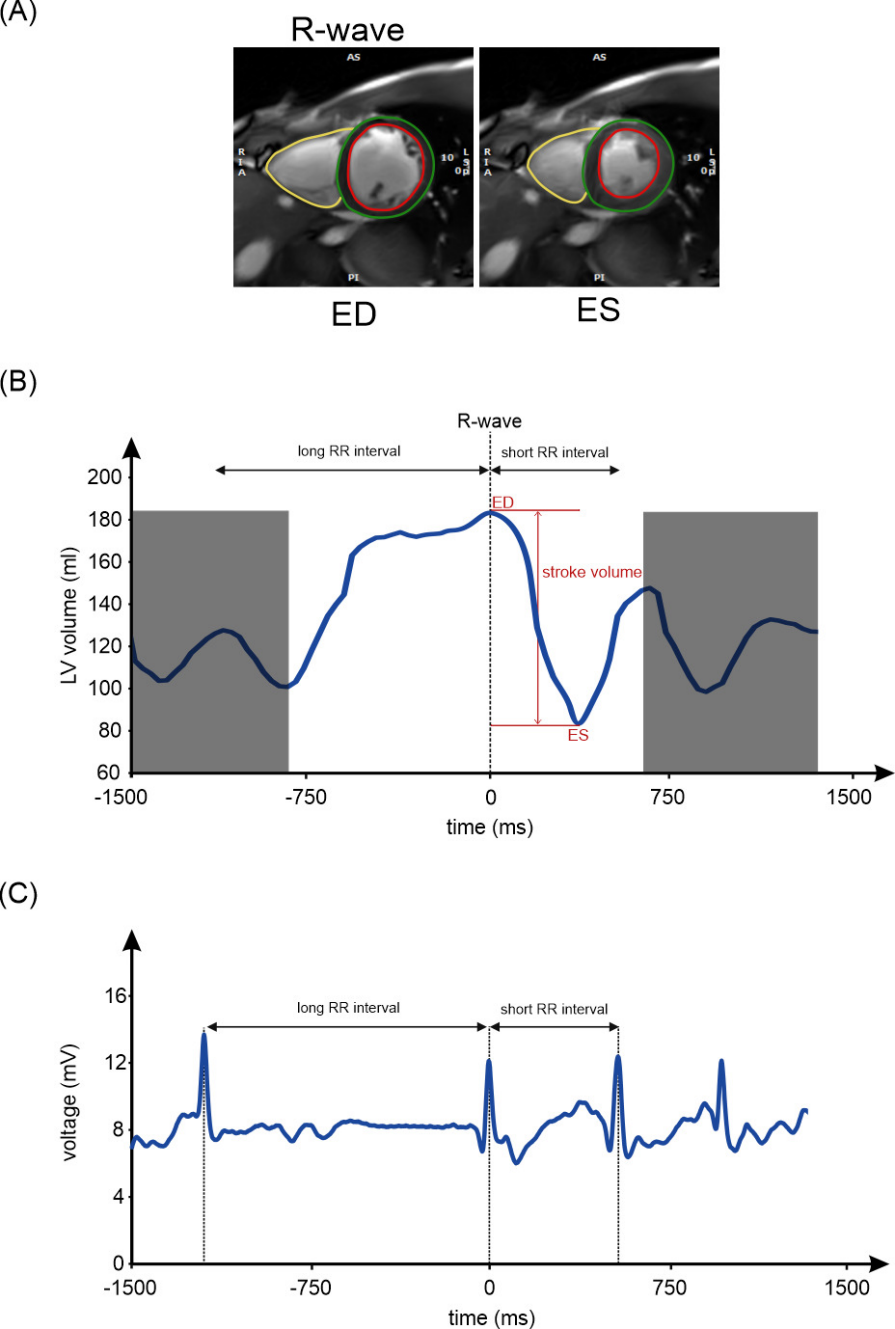
Left ventricular volumetry. (**A**) Images at end-diastole and the corresponding end-systole of a midventricular slice of the short-axis stack. (**B**) The corresponding left ventricular volume curve of the short-axis stack, with the end-diastolic phase and the corresponding end-systolic phase of all slices determining the LV stroke volume. The contractions adjacent to the sinus beat are not exactly aligned in all the slices of the short-axis stack, and therefore, the volume values cannot be evaluated and are grayed out. (**C**) The corresponding ECG of the midventricular slice shows the time of the R-wave and the corresponding long RR interval before it and the short RR interval after it, which allows for classification of the beat as a sinus beat*.* LV, left ventricular; ED, end-diastole; ES, end-systole.

The simultaneously acquired ECG was analyzed and assigned to the real-time images. This allowed the different heartbeats (sinus beats and premature beats) to be identified in each slice of the short-axis stack (Figure 2). The respiratory bellows signal was used to select heartbeats at end-expiration. In this way, it was possible to compile new short-axis stacks, each representing one of the different heartbeats (Figure 2).

Functional analysis was done using the Function SAX Module and the 2D Flow Module of commercially available analysis software [cvi42; Release 5.10.1. (1241); Circle Cardiovascular Imaging Inc., Calgary, Canada].

Real-time volumetry of a sinus beat confirmed a stable LV systolic function and stable LV volumes (Table 1). In contrast, RV function was reduced in sinus beats (Table 1). In premature beats, RV and LV end-diastolic volume, stroke volume, and ejection fraction were lower, while end-systolic volume was higher (Table 1).

**Table 1:** Left and right ventricular volumes indexed to body surface area for different heartbeats (sinus beat; premature beat class 1, i.e., a premature beat following a sinus beat; and premature beat class 2, i.e., a premature beat following another premature beat).

Cardiac volumes (indexed to BSA)	Sinus beat		Premature contraction class 1 (following a sinus beat)		Premature contraction class 2 (following a premature beat)	
	LV	RV	LV	RV	LV	RV
EDVi (ml/m^2^)	112.6 (>97th p.)	122.0 (>97th p.)	86.2 (50th–90th p.)	110.2 (50th–90th p.)	84.5 (50th–90th p.)	104.6 (50th–90th p.)
ESVi (ml/m^2^)	51.5 (>97th p.)	66.2 (>97th p.)	55.5 (>97th p.)	70.1 (>97th p.)	53.6 (>97th p.)	76.2 (>97th p.)
SVi (ml/m^2^)	61.1 (90th p.)	55.8 (50th–90th p.)	30.1 (<3rd p.)	39.7 (3rd–10th p.)	31.0 (<3rd p.)	28.3 (<3rd p.)
EF (%)	54.3	45.7	35.6	36.0	36.6	27.1

BSA, body surface area; EDVi, end-diastolic volume indexed to body surface area; ESVi, end-systolic volume indexed to body surface area; SVi, stroke volume indexed to body surface area; EF, ejection fraction; LV, left ventricle; RV, right ventricle.

**Figure 3:**
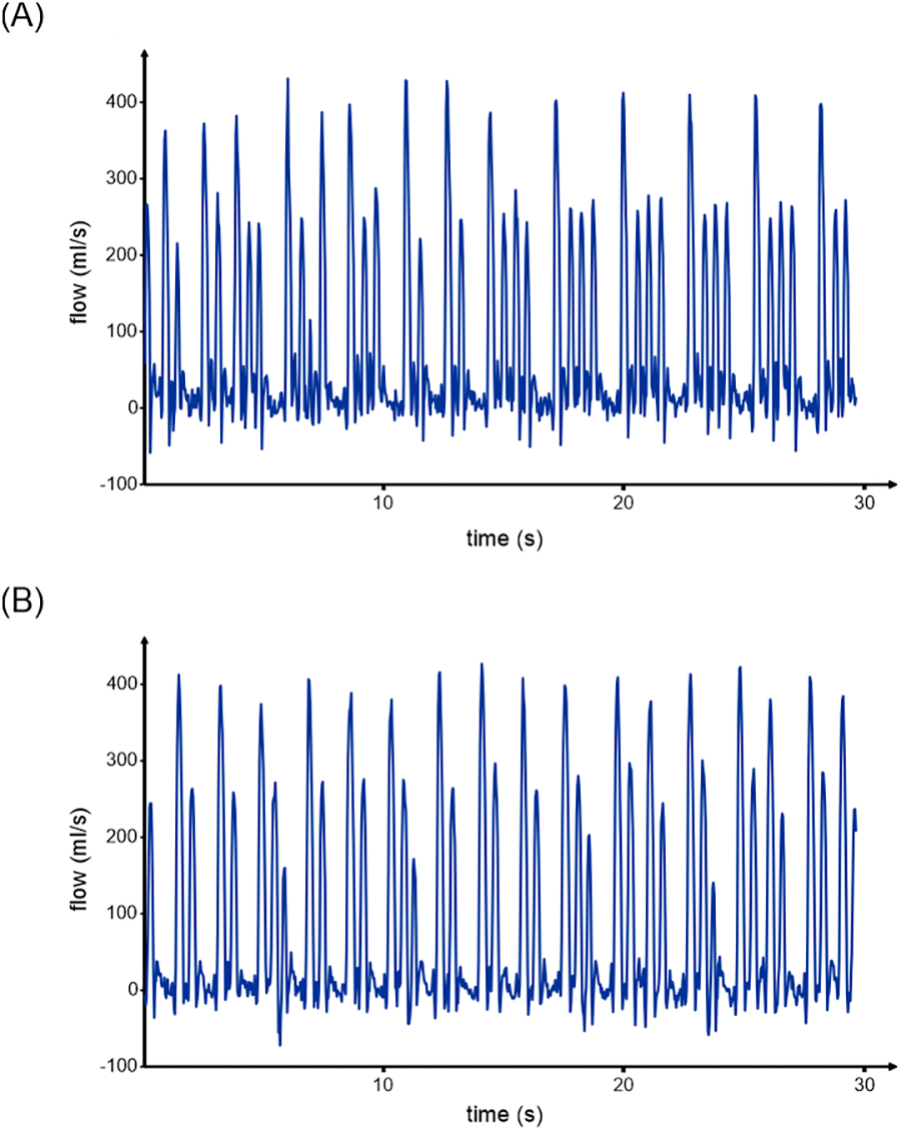
Real-time flow. (**A**) Aortic flow and (**B**) pulmonary flow over a duration of 30 s show that the amplitude after a long RR interval (sinus beat) is high, whereas the amplitude after a short RR interval (premature contraction) is low. While the amplitude of different premature beats in the aorta is similarly reduced, the amplitude of a premature contraction following a previous premature contraction in the pulmonary trunk is even lower.

Real-time aortic flow measurements over a duration of 30 s demonstrated that the antegrade flow of contractions after a long RR interval (sinus beat) was high (∼90 mL/contraction; *n* = 14), whereas the amplitude after a short RR interval (premature beat) was low (∼50 mL/contraction; *n* = 27), resulting in an average volume of 64 mL/contraction, i.e., 5.25 L/min (Figure 3).

Real-time pulmonary flow measurements showed similar results for the sinus beats (∼89 mL/contraction; *n* = 17) and the premature beats that directly followed a sinus beat (∼55 mL/contraction; *n* = 17). For premature beats that followed a previous premature beat, the antegrade flow was even more reduced (∼33 mL/contraction, *n* = 4) (Figure 3).

After an unsuccessful escalation of pharmacological therapy using flecainide, an invasive electrophysiological study conducted 23 months after the initial presentation identified an arrhythmic focus located anteroseptally on the left side of the heart, close to Bachmann's bundle, confirming the atrial origin of the extrasystoles as already suggested by the rhythmologists' interpretation of the non-invasive ECG. A second attempt to ablate it was scheduled because of an early recurrence of extrasystoles after the first ablation procedure.

## Discussion

In this case report, we showed that it is possible to use cardiac RT-MRI to obtain high-quality images of the heart in a patient with severe arrhythmia for whom conventional cardiac MRI was unable to achieve diagnostic image quality. The combination of cardiac RT-MRI and a simultaneously recorded ECG even allowed us to specifically investigate the hemodynamic consequences of both arrhythmic and rhythmic beats. While there are numerous reports on techniques to perform MR imaging despite arrhythmias (5, 14), there is a paucity of studies that attempt to analyze the arrhythmic events, use an ECG instead of the RR interval to differentiate between normal and abnormal contractions, or even differentiate and characterize different abnormal contractions, as in our case. Chava et al. applied their algorithm to one patient with premature atrial contractions (15), while Contijoch et al. tested the evaluation of each distinct beat morphology in trigeminy and bigeminy subjects (16).

The most important parameter in deciding whether a patient like ours warrants intensified antiarrhythmic therapy or a conservative observational strategy is appropriate is the risk of developing an arrhythmia-induced cardiomyopathy. Arrhythmia-induced cardiomyopathy is associated with reversible LV systolic dysfunction. This often occurs in patients with atrial fibrillation or premature ventricular complexes (17, 18). Impairment of LV function in patients with premature atrial contractions, as in our case, is rare but has also been observed (17–19).

Conventional CMR, using techniques that address the technical challenges posed by irregular heartbeat, can help detect the development of arrhythmia-induced cardiomyopathy (20). However, continuous imaging, as provided by RT-MRI, provides a more thorough understanding of the hemodynamic situation. Analyzing the hemodynamic consequences of arrhythmias, such as individual stroke volumes and ejection fractions of arrhythmic and sinus beats, along with the influence of severe arrhythmias on the cardiac dimensions, provides new diagnostic possibilities. In our case, the decrease in right ventricular stroke volume due to repetitive premature beats may be an indicator of progressive deterioration that would have been missed without continuous imaging, and it supported intensifying the antiarrhythmic therapy with the addition of a class Ic antiarrhythmic drug and subsequent invasive electrophysiological study. However, values that have prognostic significance for the development of arrhythmia-induced cardiomyopathy remain unclear at this time and will have to be investigated in further studies. This knowledge may contribute to improving counseling for these patients and their parents, who, as in our case, are frequently confronted with divergent, and occasionally even contradictory, opinions.

## Conclusion

RT-MRI allows clinicians to perform CMR even in cases with a severely irregular heart rhythm. In contrast to conventional CMR techniques for arrhythmias, which simply attempt to remove arrhythmic events from the analysis to improve image quality, RT-MRI in combination with a simultaneously acquired ECG allows for the comprehensive identification and characterization of the impact of arrhythmias on cardiovascular function and dimensions. Future studies are needed to investigate which values have prognostic significance for the development of arrhythmia-induced cardiomyopathy.

## Data Availability

The original contributions presented in the study are included in the article/Supplementary Material, further inquiries can be directed to the corresponding author.

## References

[1] Thiele H Paetsch I Schnackenburg B Bornstedt A Grebe O Wellnhofer E Improved accuracy of quantitative assessment of left ventricular volume and ejection fraction by geometric models with steady-state free precession. J Cardiovasc Magn Reson. (2002) 4(3):327–39. 10.1081/jcmr-120013298 12234104

[2] Seraphim A Knott KD Augusto J Bhuva AN Manisty C Moon JC. Quantitative cardiac MRI. J Magn Reson Imaging. (2020) 51(3):693–711. 10.1002/jmri.26789 31111616

[3] Kramer CM Barkhausen J Flamm SD Kim RJ Nagel E, Society for Cardiovascular Magnetic Resonance Board of Trustees Task Force on Standardized Protocols. Standardized cardiovascular magnetic resonance (CMR) protocols 2013 update. J Cardiovasc Magn Reson. (2013) 15(1):91. 10.1186/1532-429X-15-91 24103764 PMC3851953

[4] Ridgway JP. Cardiovascular magnetic resonance physics for clinicians: part I. J Cardiovasc Magn Reson. (2010) 12(1):71. 10.1186/1532-429X-12-71 21118531 PMC3016368

[5] Longère B Allard PE Gkizas CV Coisne A Hennicaux J Simeone A Compressed sensing real-time cine reduces CMR arrhythmia-related artifacts. J Clin Med. (2021) 10(15):3274. 10.3390/jcm10153274 34362058 PMC8348071

[6] Nacif MS Zavodni A Kawel N Choi EY Lima JA Bluemke DA. Cardiac magnetic resonance imaging and its electrocardiographs (ECG): tips and tricks. Int J Cardiovasc Imaging. (2012) 28(6):1465–75. 10.1007/s10554-011-9957-4 22033762 PMC3476721

[7] Piekarski E Chitiboi T Ramb R Feng L Axel L. Use of self-gated radial cardiovascular magnetic resonance to detect and classify arrhythmias (atrial fibrillation and premature ventricular contraction). J Cardiovasc Magn Reson. (2016) 18(1):83. 10.1186/s12968-016-0306-6 27884152 PMC5123392

[8] Voit D Zhang S Unterberg-Buchwald C Sohns JM Lotz J Frahm J. Real-time cardiovascular magnetic resonance at 1.5 T using balanced SSFP and 40 ms resolution. J Cardiovasc Magn Reson. (2013) 15(1):79. 10.1186/1532-429X-15-79 24028285 PMC3847592

[9] Frahm J Voit D Uecker M. Real-time magnetic resonance imaging: radial gradient-echo sequences with nonlinear inverse reconstruction. Invest Radiol. (2019) 54(12):757–66. 10.1097/RLI.0000000000000584 31261294

[10] Laubrock K von Loesch T Steinmetz M Lotz J Frahm J Uecker M Imaging of arrhythmia: real-time cardiac magnetic resonance imaging in atrial fibrillation. Eur J Radiol Open. (2022) 9:100404. 10.1016/j.ejro.2022.100404 35265735 PMC8899235

[11] Pettersen MD Du W Skeens ME Humes RA. Regression equations for calculation of z scores of cardiac structures in a large cohort of healthy infants, children, and adolescents: an echocardiographic study. J Am Soc Echocardiogr. (2008) 21(8):922–34. 10.1016/j.echo.2008.02.006 18406572

[12] Salameh A Gebauer RA Grollmuss O Vít P Reich O Janousek J. Normal limits for heart rate as established using 24-hour ambulatory electrocardiography in children and adolescents. Cardiol Young. (2008) 18(5):467–72. 10.1017/S1047951108002539 18634710

[13] van der Ven JPG Sadighy Z Valsangiacomo Buechel ER Sarikouch S Robbers-Visser D Kellenberger CJ Multicentre reference values for cardiac magnetic resonance imaging derived ventricular size and function for children aged 0–18 years. Eur Heart J Cardiovasc Imaging. (2020) 21(1):102–13. 10.1093/ehjci/jez164 31280290 PMC6923680

[14] Yin G Cui C An J Zhao K Yang K Li S Assessment of left ventricular systolic function by cardiovascular magnetic resonance compressed sensing real-time cine imaging combined with area-length method in normal Sinus rhythm and atrial fibrillation. Front Cardiovasc Med. (2022) 9:896816. 10.3389/fcvm.2022.896816 35711346 PMC9197321

[15] Chava R Assis F Herzka D Kolandaivelu A. Segmented radial cardiac MRI during arrhythmia using retrospective electrocardiogram and respiratory gating. Magn Reson Med. (2019) 81(3):1726–38. 10.1002/mrm.27533 30362588

[16] Contijoch F Iyer SK Pilla JJ Yushkevich P Gorman JH 3rd Gorman RC Self-gated MRI of multiple beat morphologies in the presence of arrhythmias. Magn Reson Med. (2017) 78(2):678–88. 10.1002/mrm.26381 27579717 PMC5332534

[17] Liuba I Schaller RD Frankel DS. Premature atrial complex-induced cardiomyopathy: case report and literature review. HeartRhythm Case Rep. (2020) 6(4):191–3. 10.1016/j.hrcr.2019.12.010 32322494 PMC7156972

[18] Higuchi S Kim EJ Gerstenfeld EP Bibby D Schiller NB Hsia HH. Atrial and ventricular cardiomyopathy associated with premature atrial contractions: speckle-tracking echocardiography demonstrates reversibility following successful ablation. HeartRhythm Case Rep. (2022) 8(4):243–6. 10.1016/j.hrcr.2022.01.001 35497471 PMC9039110

[19] Hasdemir C Kocabas U Kilic S Kose S Kilic S Gunduz R Premature atrial contraction-induced cardiomyopathy: recognition of a distinct phenotype of arrhythmia-induced cardiomyopathy in humans. Am J Cardiol. (2023) 197:65–7. 10.1016/j.amjcard.2023.04.016 37164875

[20] Gopinathannair R Etheridge SP Marchlinski FE Spinale FG Lakkireddy D Olshansky B. Arrhythmia-Induced cardiomyopathies: mechanisms, recognition, and management. J Am Coll Cardiol. (2015) 66(15):1714–28. 10.1016/j.jacc.2015.08.038 26449143 PMC4733572

